# Price transparency of cancer medicines: a crucial step towards informed pricing negotiations in the European region 

**DOI:** 10.3389/fphar.2026.1736741

**Published:** 2026-03-09

**Authors:** Julie Vancoppenolle, Nora Franzen, Cristina Andrianò, Morten Baltzer Houlind, Zora Ćetković, Aniek Dane, Albane Degrassat-Théas, Anne M. P. Eldering-Heldens, Martin J. Hug, Holger Knoth, Vito Ladisa, Péter Vajda, Ulrich Warnke, Valesca Retèl, Wim H. van Harten

**Affiliations:** 1 Department of Psychosocial Research and Epidemiology, Netherlands Cancer Institute-Antoni van Leeuwenhoek Amsterdam, Amsterdam, Netherlands; 2 Health Technology and Services Research Department, Technical Medical Centre, University of Twente, Enschede, Netherlands; 3 Oncological Pharmacy Unit, IRCCS Istituto Romagnolo per lo Studio dei Tumori (IRST) “Dino Amadori”, Meldola, Italy; 4 Department of Clinical Research, Copenhagen University Hospital – Amager and Hvidovre, Hvidovre, Denmark; 5 The Capital Region Pharmacy, Herlev, Denmark; 6 Department of Drug Design and Pharmacology, University of Copenhagen, Copenhagen, Denmark; 7 University Clinical Center of Serbia, Belgrade, Serbia; 8 Department of Hospital Pharmacy, Erasmus University Medical Center, Rotterdam, Netherlands; 9 Health Law Institute, INSERM UMR S 1145, Paris Cité University, Paris, France; 10 Faculty of Pharmacy, Paris Cité University, Paris, France; 11 Department of Pharmacy, Alkmaar, Netherlands; 12 Medical Center - University of Freiburg, Freiburg, Germany; 13 Pharmacy Department, University Hospital Carl Gustav Carus, Tecnical University Dresden (TUD), Dresden, Germany; 14 Departement of Hospital Pharmacy, Fondazione IRCCS Istituto Nazionale dei Tumori, Milano, Italy; 15 University Pharmacy Department of Pharmacy Administration, Semmelweis University, Budapest, Hungary; 16 Klinikum Ernst von Bergmann GmbH, Potsdam, Germany; 17 Erasmus School of Health Policy & Management Health Technology Assessment (HTA), Rotterdam, Netherlands; 18 Organization of European Cancer Institutes (OECI), Brussels, Belgium

**Keywords:** Europe, access, oncology, pricing, sustainability

## Abstract

**Background:**

The increasing cost of cancer medicines is a growing concern for hospitals in the European region, leading to cost reduction strategies, such as price negotiations. The objective of this study is to compare actual prices and discounts of cancer medicines at hospital level across the European region.

**Methods:**

To report prices and discounts without compromising the hospitals’ anonymity, an anonymization pathway was developed. Pricing data of 15 cancer medicines in 2022 was collected through structured interviews with hospital staff and reference prices were derived from the IQVIA-MIDAS dataset. Descriptive analyses were used to demonstrate product and reference prices. Discounts were aggregated across countries and product types. Furthermore, the accuracy of the participants’ perceptions of their prices compared to others within their country was assessed.

**Results:**

23 hospitals from nine countries participated. To comply with the anonymization pathway, three countries were presented in general terms. The highest prices at hospital level were observed in France, followed by Serbia, Germany, Hungary and the Netherlands. Two Southern and one Northern European country had the lowest prices. When comparing them with their reference prices, Serbia reports the lowest discounts (−7%) and the Southern European countries the highest discounts (−38%). In addition, at least half of the hospitals had incorrect perceptions of their prices relative to others within their country, with some up to 22% higher than average.

**Discussion:**

Our study is one of few providing discounts and actual prices for 15 cancer medicines, revealing large price variations between and within countries. Additionally, reference prices often fail to provide an accurate indication of the actual price and hospitals have often misguided perceptions on their negotiation performance. Transparent, standardized reporting of prices and coordinated collaborations are essential to strengthen the bargaining power of hospitals and foster informed price negotiations.

## Introduction

The share of oncology medicines entering the pharmaceutical market increased from 30% in 2010 to 50% in 2020, and its expenditure will increase from €164 billion in 2020 to €269 billion by 2025, leading to growing concern (Leufkens et al., 2022; IQVIA, 2021). The expenditure for cancer medicines leads to financial strain on healthcare systems, insurers, and even hospitals (Brinkhuis et al., 2024; Michaeli and Michaeli, 2024). In the Netherlands, the expenditure of expensive medications in hospitals has increased from €1.24 billion in 2012 to €2.64 billion in 2021, with 50% of this amount being attributed to cancer (SiRM, 2022). In response to this challenge, hospitals have been exploring cost reduction strategies to manage their pharmaceutical budget, e.g., dosage reduction, producing medicines in-house, and price negotiation strategies (Ter Heine et al., 2023; Dane et al., 2023b).

Prices and their negotiations can be opaque and fragmented within a country (Dabbous et al., 2020). Depending on the national context and product type, prices may be negotiated at national, regional, insurer, or hospital level (Boonen et al., 2010). In Italy, prices are negotiated nationally, while the prices of generics are renegotiated through public tenders at the regional level (Rossini et al., 2024; Curto et al., 2014). In the Netherlands, nationally negotiated prices can be renegotiated by insurers, hospitals and purchasing groups (SiRM, 2016). This results in multiple prices for one medicine within a country and highlights the complex pricing landscape in Europe. Confidentiality clauses further limit transparency and complicate price comparisons within and between countries (van Harten et al., 2016; De Block, 2016).

Previous research on prices of medicines is mainly based on reference prices (ex-factory prices, public retail prices) (Janssen Daalen et al., 2021). Peer-reviewed evidence on actual transaction prices, often referred to as net prices, remains scarce. Dane et al. reported that reference prices of orphan medicines varied substantially across countries, while actual prices were more homogeneous at hospital level (Dane et al., 2023a). In contrast, van Harten et al. demonstrated that actual prices of oncology medicines varied by up to 58% across hospitals in 15 European countries, with substantial differences in negotiated discounts (van Harten et al., 2016). These findings suggest that the discrepancy between reference and actual prices varies considerably depending on market structure and negotiation context.

Recent international policy developments further underscore the relevance of transparency in medicine pricing. In the United States, proposals for “most-favoured-nation” pricing seek to benchmark drug prices against those paid in comparable high-income countries, aiming to reduce prices by 30%–80% (Grueger et al., 2025; House, 2025). Such approaches rely primarily on publicly available reference prices and do not account for confidential discounts or negotiated net prices, raising concerns about distorted international price comparisons. Similar challenges are evident in Europe, where recent legislative changes, such as those under Germany’s Medical Research Act, have limited product-level price transparency. These developments have renewed calls from public interest organisations for aggregated reporting of net expenditures and discounts, consistent with WHO Resolution WHA 72.8 (Joosse et al., 2023).

Increased availability of actual price information could guide informed decision-making among hospitals and policymakers towards a sustainable pharmaceuticals market. Despite repeated calls for transparency from organisations such as the WHO and the European Parliament, progress has been limited (Joosse et al., 2023). Experimental evidence suggests that increased transparency may reduce prices without undermining incentives for research and development (Franzen et al., 2022). Improved information could also facilitate better legislation and stimulate natural experiments in this complex area. However, robust empirical evidence on actual prices at hospital level across countries remains limited.

The objective of this study is to compare actual prices and discounts of selected cancer medicines at hospital level across the European region. In addition, we examine discrepancies between actual and reference prices across countries and product characteristics (patent status, competition situation, and Anatomical Therapeutic Chemical (ATC)-4 classification). Finally, we assess the accuracy of hospitals’ perceptions of their negotiated prices relative to other hospitals within their country and region.

## Methods

This study compared prices of 15 cancer medicines across hospitals in the European region. An anonymization pathway was developed to enable publication of aggregated price data and discounts at country, ATC-4, or regional level while protecting commercially sensitive information. Pricing data referring to 2022 were collected through highly structured interviews conducted between Q3 2023 and Q2 2024, and reference prices were derived from the IQVIA MIDAS dataset (IQVIA, 2021). Descriptive analyses were used to compare actual product prices with reference prices. Sub-analyses examined discounts in relation to patent status, competition situation, and ATC-4 classification. Hospitals’ perceptions of their negotiated prices were compared with reported price levels within their country and region.

### Study design and anonymization pathway

**FIGURE 1:**
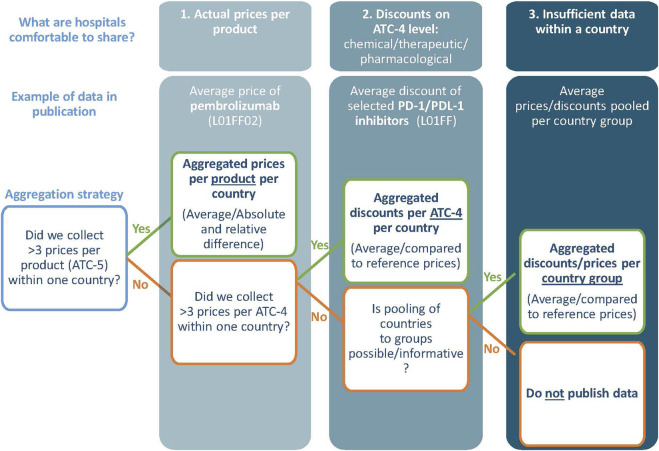
The anonymization strategy.

This cross-sectional observational study reports prices and discounts while ensuring the anonymity of hospitals. An anonymization strategy was developed under supervision of external legal counsel and followed an information exchange protocol approved by the board of the Netherlands Cancer Institute. Essentially, we report on aggregated price information following the options of our anonymization strategy (Figure 1). Preferably, at least three prices per product are collected from three hospitals per country to share the average national price (Option 1). If insufficient price data for option 1 was collected, aggregated discounts of products that have the same ATC-4 code (Option 2) and/or aggregated prices of country groups (Option 3) were reported. Additional steps were taken to increase anonymity and reduce the commercial sensitivity; e.g., price data from 2022 was transferred (and verified) orally and anonymously stored in protected portal. More details in Supplementary Appendix 1.

### Medicine selection

Medicines were selected based on relevance across hospitals and feasibility within the anonymization pathway. A pilot study involving nine hospital pharmacists from five countries (the Netherlands, Hungary, Italy, Norway, and France) identified 48 oncology medicines of interest. Selection criteria included budget impact, price level, accessibility across countries and hospital types, competition situation (monopoly, oligopoly, generic), and relevance of hospital-level negotiation. This resulted in a final selection of 15 cancer medicines across seven ATC-4 categories. Some medicines had recently passed their patent cliff in 2022 and were included due to their relevance for hospital-level renegotiations following market entry of competitors. Highly specialised treatments available only in a limited number of hospitals (e.g., cell therapies) were excluded due to anonymization constraints. It is important to note that dispensing and negotiation pathways differ across countries and products. Some included medicines may be dispensed through outpatient pharmacies in certain settings, while in others they remain hospital-based negotiated. More details in Supplementary Appendix 2.

### Data collection

Countries and hospitals were selected through purposive sampling, primarily based on feasibility of accessing confidential pricing data, existing professional networks, and requirements of the anonymization pathway. Hospitals were invited to an informational meeting where the study objectives, design, and the individual institutional requirements were presented. After agreement upon participation, a key contact person typically involved in the procurement or financial management of cancer medicines (e.g., hospital pharmacist, financial administrator) was identified. Interviews were conducted between Q3 2023 and Q2 2024 and collected retrospective pricing data from 2022. One interview was conducted per hospital (N = 23), primarily with hospital pharmacists involved in oncology medicine procurement. Interviews lasted approximately 1 hour and were conducted mainly online. A highly structured interview guide, resembling a survey instrument, was used to ensure cross-country comparability. To facilitate preparation for the interview, the interview guide was provided at least 2 weeks in advance. During the structured interview, the data was stored in an anonymized format within Castor, a secured data portal.

### Material

The structured interviews aimed to provide a comprehensive understanding on the context that influenced prices and their negotiations. General questions included topics such as the type and size of the hospital, cost reduction strategies (e.g., parallel import/export), fiscal details, and perceived price levels relative to other hospitals in the same country (higher, similar, or lower). It is important to note that timewise the perception question was asked before discussing actual prices. Medicine-specific questions focused on the prices paid by the hospital and the negotiation context. Hospitals reported the actual prices for the most frequently purchased dosage, formulation, and package size of each medicine. This approach accounted for variation in product use across hospitals and allowed capture of potential discounts related to price–volume agreements. Additionally, the context during the negotiation of a medicine, including factors like group purchases, competition situation, and indication-specific pricing were collected. More details in Supplementary Appendix 3.

### Data analysis

To calculate discounts, the actual prices reported by hospitals were compared with the corresponding reference prices. These reference prices represent publicly available list prices within each country (e.g., manufacturer or public retail prices) and do not reflect the outcomes of price negotiations or the application of discounts (Janssen Daalen et al., 2021). When comparing prices across countries, it is a challenge to select a common reference price that is comparable across countries considering differences in healthcare systems, drug distribution, and clinical practice. The IQVIA-MIDAS dataset provides different types of reference prices through periodic audits of all distribution channels within a country. It has been used previously in comparative pricing and expenditure studies as it allows for heterogeneity of healthcare systems in multi-country settings (Greiner et al., 2021; Salas-Vega et al., 2020). Due to the variability in reference prices across countries, ‘Trade Price’ was chosen as reference price as it takes the distribution structures of each country into account, ensuring comparability of the collected hospital prices across all countries and for all products. The ‘Trade Price’ was calculated based on the average sales revenue (pharmacy purchase price and wholesalers’ selling price, excluding any confidential discounts) and sold units in a year across a country. Definitions and more details in Supplementary Appendix 4.

Descriptive statistics were used to report prices and discounts at national and cross-country level excluding Value Added Tax. After data collection, actual prices were harmonized to ensure comparability across different dosages, formulations, and packaging (= Product price). The product (package size, dosage, and formulation) was selected based on the most frequent package size, dosage, and formulation collected, minimizing the need for harmonization of the actual prices. Note that reporting product prices was only possible if sufficient prices were collected to comply with the anonymization pathway. The absolute and relative differences between product prices were calculated to demonstrate price variation within and between countries. The product prices were also compared to the average reference price across all countries, allowing an international price comparison. To investigate price discrepancies, discounts were calculated using the actual and reference price and aggregated across countries and product types (patent status, market competition, and ATC-4 classification). Each hospital’s reported product prices were first aggregated and compared with the average product prices within the same country. The resulting relative price level was then contrasted with the hospital’s stated perception. Note that if potential inconsistencies were identified during data analysis, participants were contacted to reconfirm the reported price for the specific product. This verification step was used to enhance data consistency while respecting confidentiality constraints. Further details can be found in Supplementary Appendix 5, 6.

## Results

### Hospital characteristics and anonymization

Out of 82 invited hospitals, 23 hospitals from nine countries participated, including the Netherlands, France, Germany, Hungary, Serbia, a Baltic State, two Southern European countries, and one Northern European country. In the Netherlands, Germany, Hungary, and Serbia, at least three hospitals participated, allowing us to report national product prices. Although only two hospitals from France participated, national product prices could be reported as these hospitals collectively represented pricing data from more than 50 hospitals, in line with the anonymization pathway (Option 1). Prices from the Baltic State and the Northern European country could not be pooled with other countries and were therefore reported as aggregated discounts only (Option 2). Prices from three hospitals in two Southern European countries were pooled and reported at product level (Option 3). Although participants were asked about indication-specific pricing, no price differentiation was reported related to specific indications.

Most hospitals reported the use of multiple cost reduction strategies, with 22 out of 23 hospitals implementing at least one strategy. Participation in group purchasing arrangements (N = 17) and price negotiations with manufacturers or wholesalers (N = 13) were most common (Table 1). Three university hospitals reported local production of medicines, although none used this commercially. One hospital reported no active cost reduction strategies, relying exclusively on nationally negotiated prices.

**TABLE 1:** Hospital characteristics and their cost reduction strategies.

​	Hospitals (N)	Parallel import	Parallel export	Group purchasing	Precision dosing	Avoidance of medicine waste	Local production of medicines	Price (volume) negotiations	Other	No cost reduction strategies
Country										
Netherlands	6	6	0	6	5	3	1	6	4	0
Germany	4	0	0	4	0	4	2	4	3	0
Hungary	3	1	0	1	0	1	1	0	1	0
Serbia	3	0	0	0	0	0	0	0	2	1
Southern Europe (2 countries)	3	0	0	2	2	2	0	1	3	0
France	2	0	0	2	0	0	0	1	1	0
Baltic state	1	0	0	1	0	0	0	1	1	0
Northern European country	1	0	0	1	0	0	0	0	0	0
Type of hospital										
University hospital	10	2	0	7	2	3	3	6	6	0
Specialized hospital	6	1	0	4	3	2	0	1	6	0
General hospital	7	4	0	6	3	5	0	6	3	1
Pharmaceutical volume (€)										
>25 mill	3	1	0	1	0	1	0	1	1	0
25–50 mill	6	2	0	6	1	3	0	5	4	0
50–75 mill	3	2	0	2	2	1	0	2	1	0
75-100mill	5	1	0	3	2	1	0	1	5	0
100-200mill	3	0	0	2	2	3	2	2	3	1
>200mill	3	1	0	3	1	1	0	2	1	0
Total	23	7	0	17	8	10	4	13	15	1

### The differences of national product prices within and between countries

**FIGURE 2:**
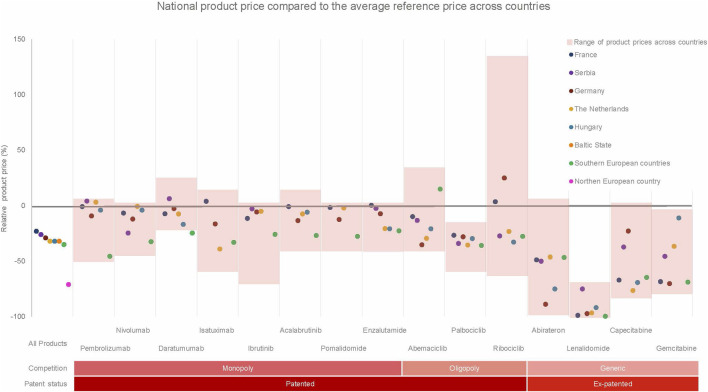
A comparison between the national product price per medicine to its average reference price for all countries from a hospital perspective. The red boxes indicate the price range observed for every product across all countries while safeguarding anonymity of the country. The Northern EU country only shared prices for the medicines in an oligopoly or generic situation.

Substantial variation in product prices was observed both within and between countries. Table 2 presents national and cross-country product prices and their relative and absolute differences. Figure 2 compares national product prices with the average reference price across all participating countries.

**TABLE 2:** National product price and reference price with absolute and relative differences from a hospital perspective.

Country/Region		All countries (N = 23)			Netherlands (N = 6)			Germany (N = 4)			Serbia (N = 3)			Hungary (N = 3)			Southern Europe (N = 3)		
GDP per capita (2023)		39,525			43,420			35,630			7736			20,648			20,000–40000		
Medicine	Price (Euro)	Average (€)	Abs diff (€)	Rel.Diff (%)	Average (€)	Abs diff (€)	Rel.Diff (%)	Average (€)	Abs diff (€)	Rel.Diff (%)	Average (€)	Abs diff (€)	Rel.Diff (%)	Average (€)	Abs diff (€)	Rel.Diff (%)	Average (€)	Abs. Diff(€)	Rel. Diff (%)
ABEMACICLIB	Product price	1875	2,274	121	1,605	1,376	86	1,472	128	9	1974	0	0	​	​	​	2,615	1,008	39
*FC tab 150mgx56*	Reference price	2,360	​	​	2,404	​	​	1,472	​	​	1990	​	​	1935	​	​	3,789	​	​
RIBOCICLIB	Product price	2,161	3,961	183	1852	413	22	3,016	3,791	126	1756	78	4	​	​	​	1749	340	19
*FC tab 200 mg x 63*	Reference price	2,593	​	​	2007	​	​	2,398	​	​	1852	​	​	1745	​	​	4,559	​	​
PALBOCICLIB	Product price	1702	653	38	1,618	408	25	1800	547	30	1,651	517	31	​	​	​	1,607	544	34
*FC tab 125 mg x 21*	Reference price	2,696	​	​	1976	​	​	1962	​	​	1960	​	​	1938	​	​	3,600	​	​
ACALABRUTINIB	Product price	4,488	2,676	60	​	​	​	​	​	​	​	​	​	​	​	​	3,801	1,538	40
*Cap 100 mg x 56*	Reference price	5,242	​	​	4,840	​	​	4,758	​	​	​	​	​	5,279	​	​	5,244	​	​
IBRUTINIB	Product price	4,368	3,597	82	4,896	1,225	25	4,873	328	7	5,019	25	0	​	​	​	​	​	​
*FC tab 420 mg x30*	Reference price	5,124	​	​	5,810	​	​	5,060	​	​	5,107	​	​	4,563	​	​	5,475	​	​
DARATUMUMAB	Product price	4,278	1,138	27	4,358	36	1	4,584	260	6	5,011	26	1	​	​	​	​	​	​
*INF 120 mg/ml 15 mL*	Reference price	4,638	​	​	4,887	​	​	4,693	​	​	5,061	​	​	4,140	​	​	5,101	​	​
ISATUXIMAB	Product price	2,311	2,168	94	1801	1,273	71	2,472	1,664	67	​	​	​	​	​	​	1980	2030	102
*INF 20 mg/ml 25 mL*	Reference price	2,819	​	​	2,452	​	​	2,797	​	​	​	​	​	​	​	​	2,885	​	​
PEMBROLIZUMAB	Product price	2,265	1,381	61	2,611	26	1	2,294	184	8	2,634	13	0	​	​	​	1,379	407	30
*INF 25 mg/ml 4 mL*	Reference price	2,661	​	​	2,640	​	​	2,315	​	​	2,659	​	​	2,330	​	​	1,344	​	​
NIVOLUMAB	Product price	961	514	53	1,077	99	9	952	138	15	817	296	36	​	​	​	733	404	55
*INF 10 mg/ml 10 mL*	Reference price	1,102	​	​	1,019	​	​	978	​	​	1,029	​	​	1,157	​	​	8,885	​	​
POMALIDOMIDE	Product price	6,955	3,098	45	7,651	711	9	6,845	1,076	16	​	​	​	​	​	​	5,653	2,642	47
*Cap 4 mg x21*	Reference price	7,787	​	​	8,723	​	​	7,346	​	​	​	​	​	6,325	​	​	8,885	​	​
LENALIDOMIDE	Product price	137	927	677	174	927	534	141	300	213	1,250	6	0	​	​	​	19	8	44
*Cap 25 mg x21*	Reference price	6,073	​	​	5,513	​	​	3,633	​	​	1,172	​	​	4,032	​	​	5,818	​	​
ENZALUTAMIDE	Product price	2,389	1,246	52	2,241	217	10	2,619	317	12	2,754	14	0	​	​	​	2,185	1,088	50
*FC tab 40 mg x112*	Reference price	2,678	​	​	2,897	​	​	2,851	​	​	2,780	​	​	2,387	​	​	3,405	​	​
ABIRATERONE	Product price	1,076	2,841	264	1,447	2,841	196	300	716	239	1,348	7	0	​	​	​	1,435	2,375	166
*FC tab 500 mg x56*	Reference price	2,678	​	​	2,445	​	​	3,008	​	​	2,918	​	​	2,243	​	​	3,243	​	​
CAPECITAB1NE	Product price	45	99	219	28	9	30	93	70	75	75	1	2	37	30	82	42	39	93
*FC 500 mg x120*	Reference price	127	​	​	153	​	​	138	​	​	77	​	​	88	​	​	121	​	​
GEMCITAB1NE	Product price	29	44	151	36	30	84	17	1	3	31	4	12	50	5	10	18	15	87
*INF 100 mg/ml 20 mL*	Reference price	61	​	​	71	​	​	22	​	​	30	​	​	57	​	​	129	​	​

That some national product prices could not be reported as less than three prices were obtained within a country (group).

Across countries, the largest variation was observed for ribociclib (absolute and relative difference: €3,961; 183%), followed by pomalidomide (€3,098; 45%). The smallest variation was observed for daratumumab (€1,138; 27%) and palbociclib (€653; 38%).

Compared with average reference prices, France reported the highest average product prices (−23%), followed by Serbia (−26%), Germany (−29%), Hungary (−32%), and the Netherlands (−32%). The two Southern European countries reported lower prices (−35%), while the Northern European country reported the lowest prices (−72%), although prices in the latter were only available for oligopoly and generic medicines.

Within-country variation was also observed. In the Netherlands, large differences were found for abiraterone and lenalidomide, whereas Germany showed substantial variation for ribociclib and abiraterone. Serbia showed more limited within-country variation.

### Discounts reported per patent status, competition situation and ATC-4 level

**FIGURE 3:**
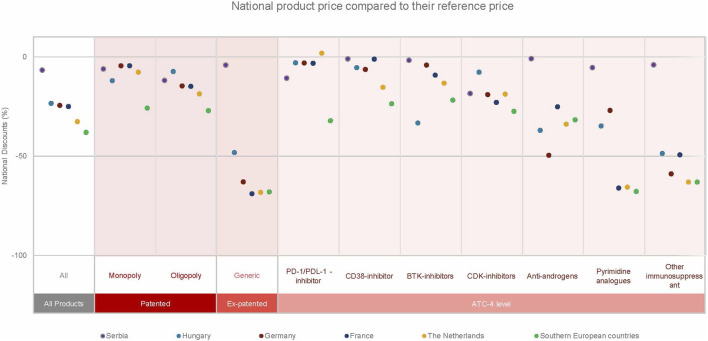
Discounts aggregated across countries and product types (patent status, competition situation and ATC-4 level).

Figure 3 presents discounts aggregated across countries and product characteristics. Discount levels varied substantially across countries and market structures. Serbia reported the lowest overall discount (−7%), followed by Hungary (−23%), while the Southern European countries reported the highest discounts (−38%), followed by the Netherlands (−33%).

For monopoly medicines, discounts were lowest in France and Germany and higher in Hungary and the Southern European countries. For oligopoly medicines, the largest discounts were observed in the Netherlands, France, and Germany. Generic medicines showed the highest discounts overall, particularly in France, the Netherlands, and the Southern European countries, while Serbia reported minimal discounts for generics.

At ATC-4 level, PD-1/PD-L1 inhibitors showed consistently low discounts across countries, whereas other immunosuppressants showed higher discounts, except in Serbia.

### Comparison between the hospitals’ perceived price levels compared to the reported price levels

**FIGURE 4:**
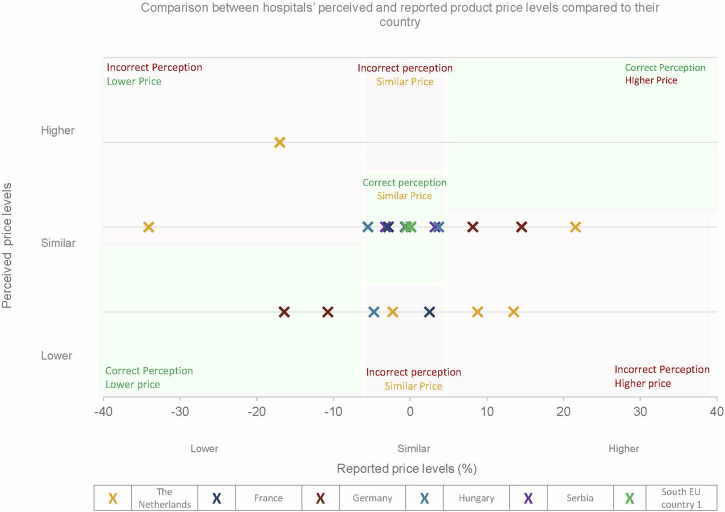
Assessing whether participants’ perception of their prices relative to others within their country are (in) correct. A difference of 5% from the average price level was chosen as a cut-off point for the similar price level. Note that in France, only two hospitals were included.

Figure 4 shows the hospitals’ perceived price levels compared to reported prices within their country. It is important to note that for France, only two respondents participated in the analysis. However, these respondents represent over 50 hospitals. Hospitals’ perceptions of their negotiated prices frequently did not align with reported price levels. Among 20 hospitals included in this analysis, ten had incorrect perceptions. Several hospitals perceived their prices as lower than those of peers, while their reported prices were up to 22% higher than national averages. Similar misperceptions were observed when comparing hospitals’ perceived prices to regional averages. More details in Supplementary Appendix 7.

## Discussion

In this study, we provide insights into discounts and actual prices from 23 hospitals across nine countries. Significant variations in product prices were observed between and within countries. The highest average product prices at hospital level were reported in France and Serbia and the lowest product prices in the Southern European countries and the Northern European country. In all countries, large price differences within a country existed, except for Serbia. When comparing the actual prices to their national reference prices, the lowest national discount was observed in Serbia (−7%) and the highest in the Southern European countries (−38%). In addition, at least half of the hospitals had incorrect perceptions of their negotiation performance, with some having higher prices up to 22% than their national- and 30% to their regional average.

Variations in product and reference prices across countries are to be expected (Vogler et al., 2016). However, our study reveals that countries with lower GDP *per capita* do not consistently benefit from lower product prices, despite of their lower reference prices. For instance, the product price for abemaciclib was the highest in Serbia and the lowest in the Netherlands and Germany. Serbia also paid the highest prices for other products like ibrutinib, daratumumab, lenalidomide, and enzalutamide. These findings align with previous research, which indicated that countries with higher GDP *per capita*, often secure lower medicine prices, even when adjusted for purchasing power parity (PPP) (Moye-Holz and Vogler, 2022). Although our study did not adjust for PPP, it supports previous research highlighting the disconnect between prices and GDP *per capita* (Chapman et al., 2020; Hawlik K, 2014). A plausible explanation lies in structural market dynamics. Lower-GDP countries typically face tighter health budgets, reduced market attractiveness, and weaker bargaining power, which together limit their ability to negotiate favorable prices despite lower reference prices (Büssgen and Stargardt, 2022; Vogler et al., 2012).

Product price comparisons should be approached with caution due to national price regulations, fragmented pricing and varying access to price information. For instance, while nationally negotiated prices may be available to hospitals in some countries, this is not the case in others. In our study, nationally negotiated prices were reported for certain products in Hungary, one Southern European country, and one Northern European country, but legal constraints prevented their disclosure, requiring aggregation. In countries like France, aggregated national negotiation outcomes are published, but hospitals are not informed about their negotiated prices (Comité économique des produits de santé: rapport d’activité, 2022). In some countries, access to pricing information varies also by product type. In Germany, the prices of medicines dispensed in pharmacies are publicly available, whereas prices for hospital medicines are not. This fragmentation and opacity complicates informed negotiations for hospitals and hinder direct price comparisons across countries.

Large price variation across products was observed in all countries except Serbia, where prices are predominantly negotiated at the national level. This variation was closely linked to market structure. Medicines in markets where hospitals had greater bargaining power, particularly oligopolistic markets, exhibited higher discounts and greater price dispersion, whereas patented medicines in strict monopoly settings showed the lowest discounts and the most limited variation. This pattern was especially evident in the Netherlands and Germany, where hospital pharmaceuticals are commonly procured through hospital-level or group-based negotiations, resulting in confidential discounts and price variation, particularly in markets with limited competition (SiRM, 2016). In contrast, for ex-patented medicines, particularly following patent expiry, substantial within-country price variation emerged, contributing to pronounced price differences for products such as lenalidomide and abiraterone. These findings underscore the role of competition in strengthening bargaining power and highlight the need for critical scrutiny of patent-related practices, including evergreening, that may delay effective market competition.

This study revealed significant discrepancies between reference prices and actual product prices, with reference prices often failing to provide an accurate indication of the actual price at hospital level. This was observed across all countries, whereas reference prices were least reflective of actual prices in the two Southern European countries. In contrast, Serbia’s reference prices provided a somewhat better indication of prices at hospital level, although even there, discrepancies existed. These findings underscore a key limitation of reference prices as they fail to capture the effects of confidential negotiations and discounts. As a result, reliance on reference prices in budget impact analyses or external reference pricing may lead to distorted cost estimates and suboptimal policy decisions.

These limitations of reference pricing extend beyond the European region and reflect a broader structural challenge in pharmaceutical pricing policy. In the United States, the proposed “most-favored-nation” model similarly relies on international reference pricing derived from list prices and does also not account for confidential discounts and negotiated rebates. As in the European context examined here, this approach risks systematically overestimating actual prices and may incentivize strategic behaviors such as list price inflation or reduced transparency. This paradox highlights that reference pricing, when detached from transaction prices and unaccompanied by harmonized reporting mechanisms, can increase opacity, with unclear implications for affordability. Consequently, greater alignment between reference and actual prices could improve cross-country comparisons and increase the effectiveness of joint health technology assessment and coordinated pharmaceutical policy initiatives aimed at pharmaceutical sustainability. Further research is needed to determine the appropriate level of anonymized, aggregated price transparency that balances affordability, equity, market efficiency, and competition, while supporting equitable access to medicines and sustaining Europe’s attractiveness for pharmaceutical investment and innovation.

This tension between the growing emphasis on transparency and persistent information asymmetries is reflected in the participation barriers encountered in this study. While hospitals expressed strong interest in the research and acknowledged its relevance, participation was nevertheless constrained by a combination of confidentiality obligations, regulatory and strategic considerations, as well as financial dependencies on pharmaceutical arrangements (e.g., financing clinical trials). These barriers, together with widespread misperceptions about negotiation performance and substantial price variation within and across countries, underscore the need for greater transparency and collaboration in price negotiations. At the same time, the heterogeneity across providers, products, and national contexts continues to complicate the identification of transferable best practices.

Several recommendations can enhance informed price negotiations and bargaining power. First, developing standardized reporting guidelines and sharing aggregated price information could help hospitals evaluate their negotiation performance and adjust strategies. For instance, France’s “Enquête Achat et Consommation de Médicaments à l'Hôpital” and the Netherlands’ aggregated pricing reports from insurers exemplify how such reports, though not publicly disclosed, assist hospitals in negotiations. Expanding the legal framework for anonymizing and aggregating pricing data could facilitate the sharing of price information while ensuring compliance with confidentiality clauses, improving hospital’s knowledge on their performance and identifying medicines with greater bargaining potential. Second, adopting unified negotiation strategies can strengthen bargaining positions. Standardizing negotiation protocols across hospitals could enhance tendering processes, while regular price negotiations would ensure prices reflect current market conditions. For example, many contracts for oligopoly drugs in the Netherlands include clauses allowing price negotiations to restart when market conditions change. Our also findings suggest that transferable elements such as centralized negotiation structures and competitive tendering frameworks may contribute to higher discounts, although institutional differences limit direct policy transferability. Finally, natural experiments with increased transparency can further refine these strategies.

Several limitations should be considered when interpreting the findings of this study. Variations in participation and pricing data collection, largely driven by legal and institutional constraints, limited data completeness and may have introduced response bias. Participation barriers were primarily structural, including confidentiality obligations and fragmented ownership of price information. For example, the United Kingdom and Norway declined participation due to the absence of institutional responsibility for hospital-level prices. In the absence of reliable national benchmark data, it is also not possible to assess country performance accurately. Comparability across countries is further constrained by differences in negotiation arrangements, such as managed entry agreements. Actual prices may thus incorporate nationally negotiated discounts by public authorities, which are not always visible to hospitals. In France, for instance, hospitals directly negotiated prices for only six of the 15 selected medicines, while the remaining products were subject to national-level negotiations, leaving hospitals without access to confidential price information. In addition, some medicines were obtained under derogatory access schemes, allowing temporary free price setting prior to completion of the full market access process. For these products, legally mandated discounts negotiated by the Comité Économique des Produits de Santé, such as annual and settlement discounts, apply once reimbursement is granted. Moreover, detailed information on specific agreements, including price–volume arrangements and clawbacks, could not be fully collected. Caution is warranted when interpreting findings related to purchase volumes, as volumes do not necessarily reflect hospital bargaining power but may instead be driven by clinical need. Finally, although participants considered the reported data to be accurate and potential irregularities were verified where possible, some risk of reporting error remains. Despite these limitations, this study documents substantial variation in actual prices and discounts for 15 cancer medicines at hospital level, using innovative strategies to safeguard the confidentiality of participating hospitals.

## Conclusion

This study involving 23 hospitals from nine European countries reveals significant price variations of 15 cancer medicines between and within countries. Reference prices were frequently poor proxies for actual transaction prices, and hospitals often misjudged their relative negotiation performance. Transparent and standardized reporting of prices and coordinated collaborations among hospitals and countries are essential to strengthen the bargaining power of hospitals and foster informed price negotiations.

## Data Availability

The datasets presented in this article are not readily available because Author elects to not share less aggregated data than presented in this study. Research data are not shared due to its sensitive and confidential nature. Requests to access the datasets should be directed to w.h.vanharten@nki.nl.

## References

[B1] Boonen L. H. Van Der Geest S. A. Schut F. T. Varkevisser M. (2010). Pharmaceutical policy in the Netherlands: from price regulation towards managed competition. Adv. Health Econ. Health Serv. Res. 22, 53–76. 10.1108/s0731-2199(2010)0000022006 20575228

[B2] Brinkhuis F. Goettsch W. G. Mantel-Teeuwisse A. K. Bloem L. T. (2024). High cost oncology drugs without proof of added benefit are burdening health systems. BMJ 384, q511. 10.1136/bmj.q511 38423553

[B3] Büssgen M. Stargardt T. (2022). Changes in launch delay and availability of pharmaceuticals in 30 European markets over the past two decades. BMC Health Serv. Res. 22, 1457. 10.1186/s12913-022-08866-7 36451186 PMC9714155

[B4] Chapman S. Paris V. Lopert R. (2020). Challenges in access to oncology medicines.

[B26] Comité économique des produits de santé: rapport d’activité (2022). Comité économique des produits de santé: rapport d’activité 2022.

[B5] Curto S. Ghislandi S. Van De Vooren K. Duranti S. Garattini L. (2014). Regional tenders on biosimilars in Italy: an empirical analysis of awarded prices. Health Policy 116, 182–187. 10.1016/j.healthpol.2014.02.011 24602376

[B6] Dabbous M. Chachoua L. Caban A. Toumi M. (2020). Managed entry agreements: policy analysis from the european perspective. Value Health 23, 425–433. 10.1016/j.jval.2019.12.008 32327159

[B7] Dane A. Klein Gebbink A.-S. Brugma J.-D. Degrassat-Théas A. Hug M. J. Houlind M. B. (2023a). Prices of orphan drugs in four Western European countries before and after market exclusivity expiry: a cross-country comparison of list prices and purchase prices. Appl. Health Econ. Health Policy 21, 905–914. 10.1007/s40258-023-00832-6 37751107 PMC10628053

[B8] Dane A. Van Leeuwen R. Hoedemakers M. Van Der Kuy H. Sleijfer S. (2023b). Combatting the rising costs of cancer drugs; interventions from a university hospital's perspective. Front. Pharmacol. 14, 1264951. 10.3389/fphar.2023.1264951 37701038 PMC10493871

[B9] De Block M. (2016). The difficulty of comparing drug prices between countries. Lancet Oncol. 17, e125. 10.1016/S1470-2045(16)00176-5 27300660

[B11] Franzen N. Ziegler A. Romagnoli G. Retèl V. P. Offerman T. J. S. Van Harten W. H. (2022). Affordable prices without threatening the oncological R&D Pipeline-An economic experiment on transparency in price negotiations. Cancer Res. Commun. 2, 49–57. 10.1158/2767-9764.CRC-21-0031 36860697 PMC9973423

[B12] Greiner W. Patel K. Crossman-Barnes C. J. Rye-Andersen T. V. Hvid C. Vandebrouck T. (2021). High-expenditure disease in the EU-28: does drug spend correspond to clinical and economic burden in oncology, autoimmune disease and diabetes? Pharmacoecon Open 5, 385–396. 10.1007/s41669-020-00253-4 33411314 PMC8333173

[B13] Grueger J. Martin K. Sullivan S. D. (2025). Referencing drug prices of other countries may not sustainably lower prices in the United States: lessons from Europe. Value Health 28, 1305–1308. 10.1016/j.jval.2025.06.010 40609636

[B14] Hawlik K. D. A. (2014). Access to high-priced medicines in hospital settings in Europe: a study in four European countries health Action international.

[B15] House T. W. (2025). Delivering most-favored-nation prescription drug pricing to American patients. Available online at: https://www.whitehouse.gov/presidential-actions/2025/05/delivering-most-favored-nation-prescription-drug-pricing-to-american-patients/ (Accessed December 12, 2025).

[B16] IQVIA (2021). Global oncology trends 2021: outlook on 2030.

[B17] IQVIA MIDAS (2022). All rights reserved.

[B18] Janssen Daalen J. M. Den Ambtman A. Van Houdenhoven M. Van Den Bemt B. J. F. (2021). Determinants of drug prices: a systematic review of comparison studies. BMJ Open 11, e046917. 10.1136/bmjopen-2020-046917 34266841 PMC8287090

[B19] Joosse I. R. Tordrup D. Glanville J. Kotas E. Mantel-Teeuwisse A. K. Van Den Ham H. A. (2023). Evidence on the effectiveness of policies promoting price transparency - a systematic review. Health Policy 134, 104681. 10.1016/j.healthpol.2022.11.002 36372608 PMC10357344

[B20] Leufkens H. G. Kusynová Z. Aitken M. Hoekman J. Stolk P. Klein K. (2022). Four scenarios for the future of medicines and social policy in 2030. Drug Discov. Today 27, 2252–2260. 10.1016/j.drudis.2022.03.018 35364271

[B21] Michaeli D. T. Michaeli T. (2024). Launch and post-launch prices of injectable cancer drugs in the US: clinical benefit, innovation, epidemiology, and competition. Pharmacoeconomics 42, 117–131. 10.1007/s40273-023-01320-4 37855850 PMC10791980

[B22] Moye-Holz D. Vogler S. (2022). Comparison of prices and affordability of cancer medicines in 16 countries in Europe and Latin America. Appl. Health Econ. Health Policy 20, 67–77. 10.1007/s40258-021-00670-4 34228312 PMC8752537

[B23] Rossini E. E. Galeone C. Lucchetti C. Jommi C. (2024). From indication-based pricing to blended approach: evidence on the price and reimbursement negotiation in Italy. Pharmacoecon Open 8, 251–261. 10.1007/s41669-023-00467-2 38228997 PMC10883902

[B25] Salas-Vega S. Shearer E. Mossialos E. (2020). Relationship between costs and clinical benefits of new cancer medicines in Australia, France, the UK, and the US. Soc. Sci. and Med. 258, 113042. 10.1016/j.socscimed.2020.113042 32480184

[B27] SIRM (2016). Versterken inkoop geneesmiddelen: onderzoek in opdracht van de Nederlandse Vereniging van Ziekenhuizen.

[B28] SIRM (2022). Prognose uitgaven add-on geneesmiddelen 2022-2026.

[B29] Ter Heine R. Van Den Heuvel M. M. Piet B. Deenen M. J. Van Der Wekken A. J. Hendriks L. E. L. (2023). A systematic evaluation of cost-saving dosing regimens for therapeutic antibodies and antibody-drug conjugates for the treatment of lung cancer. Target Oncol. 18, 441–450. 10.1007/s11523-023-00958-6 37081309 PMC10192147

[B30] Van Harten W. H. Wind A. De Paoli P. Saghatchian M. Oberst S. (2016). Actual costs of cancer drugs in 15 European countries. Lancet Oncol. 17, 18–20. 10.1016/S1470-2045(15)00486-6 26670093

[B31] Vogler S. Zimmermann N. Habl C. Piessnegger J. Bucsics A. (2012). Discounts and rebates granted to public payers for medicines in European countries. South Med. Rev. 5, 38–46. Available online at: https://pubmed.ncbi.nlm.nih.gov/23093898/. 23093898 PMC3471187

[B32] Vogler S. Vitry A. Babar Z.-U.-D. (2016). Cancer drugs in 16 European countries, Australia, and New Zealand: a cross-country price comparison study. Lancet Oncol. 17, 39–47. 10.1016/S1470-2045(15)00449-0 26670089

