# Supplementary material for: Price transparency of cancer medicines: a crucial step towards informed pricing negotiations in the European region
Source: Front Pharmacol. 2026 Mar 9;17:1736741. doi: 10.3389/fphar.2026.1736741 (PMC13006507; doi:10.3389/fphar.2026.1736741)
Supplement: Supplementary file 1 [file Supplementaryfile1.docx]

**Appendices**

Contents

[Appendix 1: Ethical and legal considerations 2](#_Toc190520592)

[Appendix 2: Selection of medicines 3](#_Toc190520593)

[Appendix 3: The interview guide 5](#_Toc190520594)

[Appendix 4: Choosing the right comparator 11](#_Toc190520595)

[Appendix 5: Price harmonization 13](#_Toc190520596)

[Appendix 6: Data analysis 14](#_Toc190520597)

[Appendix 7: Hospitals’ perceived price levels compared to the reported price levels within their European region 16](#_Toc190520598)

# Appendix 1: Ethical and legal considerations

It is well known that exchanging information on pharmaceutical prices is sensitive in terms of confidentiality and competition law. Therefore, we have consulted external legal counsel to ensure that our methods are compliant with the sensitivity of the data.

We have taken and will take a number of steps to maximize confidentiality:

1. Confidential Information submitted to the EFPN will be anonymized and aggregated before any publication or broadcasting so that it cannot be traced back to one single institute, hospital, organisation or patient or a limited number of those.
2. Price data will be at least one year old at the time of publication. Therefore prices are collected from 2022.
3. The research division of EFPN will act as the primary and only recipient of Confidential Information within EFPN. Data will only be shared by members on a predefined insider list.
4. Data will be transferred orally and stored anonymously in a protected portal (Castor).
5. The research division will process Confidential Information by secured e-mailing. The research division will store information, including Confidential Information, in a secure and confidential filing location, with sole access for researchers on the insider list.

# Appendix 2: Selection of medicines

The selection of medicines for this study was guided by consideration of relevancy and anonymization. In order to compose a list of anticancer medicines that is relevant across hospital types and European countries, and to enhance the comprehensiveness of our study, medicines were selected through the pilot study involving representatives from nine hospitals across five countries (NL, HU, IT, NO, FR). The participants, mainly hospital pharmacists, provided their top 10 list of medicines and feedback on the relevance of negotiation of the medicines within their specific national and hospital settings. From this preliminary investigation, 48 cancer medicines of interest were identified. The following selection criteria guided our assessment of relevancy: oncology or hematology, high budget impact, high price, accessibility across hospital types and countries, competition (monopoly/oligopoly/generic), significance of negotiations at hospital level. This resulted in the inclusion of 15 anticancer medicines of seven ATC-level 4 categories from nine pharmaceutical companies. Importantly, next two relevancy, the selection of medicines was also guided by the anonymization pathway. As an implication, we were not allowed to add treatments that are only available in few specialized hospitals across Europe (such as Cell therapies). Furthermore, two additional medicines were included to reinforce the anonymization strategy on ATC-4 level, ensuring the integrity and ethical conduct of our subsequent analyses.

| Reason inclusion | ATC-5 | Medicine | ATC-4 |  | ATC-3 | Votes | Brandname | Adm. | Competition | Generic available | Ema approval | MAH | treatment |
| --- | --- | --- | --- | --- | --- | --- | --- | --- | --- | --- | --- | --- | --- |
| Oligopoly situation | L01EF01 | Palbociblib | L01EF | CDK-inhibitors | L01E | 2 | Ibrance | O | Oligopoly | no | 2016 | Pfizer | Breast cancer |
| Oligopoly situation | L01EF02 | Ribociclib | L01EF | CDK-inhibitors | L01E | 2 | Kisqali | O | Oligopoly | no | 2017 | Novartis | Breast cancer |
| Oligopoly situation | L01EF03 | Abemaciclib | L01EF | CDK-inhibitors | L01E | 2 | Verzenios | O | Oligopoly | no | 2018 | Eli Lilly | Breast cancer |
| interest of hospital | L01EL01 | Ibrutinib | L01EL | BTK-inhibitors | L01E | 4 | Imbruvica | O | monopoly | no | 2014 | AbbVie and Janssen | CLL |
| Anonymisation+interest | L01EL02 | Acalabrutinib | L01EL | BTK-inhibitors | L01E | 2 | Calquence | O | monolopy | no | 2017 | AstraZeneca | CLL |
| interest of hospital | L01FC01 | Daratumumab | L01FC | CD38 -inhibitors | L01F | 3 | Darzalex | IV | Monopoly | yes | 2016 | Janssen Biotech | Multiple meyloma |
| Anonymisation | L01FC02 | Isatuximab | L01FC | CD38 -inhibitor | L01F | 0 | Sarclisa | IV | monopoly? | no | 2020 | Sanofi | Multiple meyloma |
| Price and budget impact+ analysis of monopoly | L01FF01 | Nivolumab | L01FF | PD-1/PDL-1 -inhibitor | L01F | 1 | Opdivo | IV | Monopoly | no | 2015 | Bristol-Myers Squibb | multiple |
| Price and budget impact+ analysis of monopoly | L01FF02 | Pembrolizumab | L01FF | PD-1/PDL-1 -inhibitor | L01F | 1 | Keytruda | IV | Monopoly | no | 2015 | Merck | multiple |
| Anonymisation | L02BB04 | Enzalutamide | L02BB | Anti-androgens | L02B | 3 | Xtandi | O | Oligopoly? | no | 2013 | Pfizer and Astellas | Prostate cancer |
| Price drop | L02BX03 | Abiraterone | L02BX | [Other hormone antagonists and related agents](https://www.whocc.no/atc_ddd_index/?code=L02BX&showdescription=no) | L02B | 3 | Zytiga | O | Generic | yes | 2011 | Janssen Biotech | Prostate cancer |
| Price drop | L04AX04 | Lenalidomide | [L04AX](https://www.whocc.no/atc_ddd_index/?code=L04AX&showdescription=no) | Other immunosuppressants | L04A | 3 | Revlimid | O | Generic | yes | 2007 | Celgene (now part of Bristol-Myers Squibb) | multiple meylanoma |
| Anonymisation | L04AX06 | Pomalidomide | [L04AX](https://www.whocc.no/atc_ddd_index/?code=L04AX&showdescription=no) | Other immunosuppressants | L04A | 1 | Pomalyst | O | Monopoly | no | 2013 | Celgene (now part of Bristol-Myers Squibb) | multiple meylanoma |
| Increase country relevance | L01BC05 | Gemcitabine | L01CB | Pyrimidine analogues | L01C | 1 | Gemzar | IV | Generic | yes | 1998 | Bristol Myers Squibb | Breast cancer, ovarian cancer, lung cancer |
| Increase country relevance | L01BC06 | Capecitabine | L01CB | Pyrimidine analogues | L01C | 1 | Xeloda | O | Generic | yes | 2002 | Celgene Corporation (now Bristol Myers Squibb) | Colorectal and breast cancer |

# Appendix 3: The interview guide

*General questions*

1. In which country is your hospital located?
2. What is your type of hospital?

- University hospital
- Specialized cancer hospital
- General hospital
- Other: Which type?___________________

1. How much does your hospital approximately spend on buying pharmaceutical products annually?

- <25 million euros
  - If this: how much?
- 25-50 million euros
- 50-75 million euros
- 75-100million euros
- 100-125million euros
- 125-150 million euros
- 150-175 million euros
- 175-200 million euros
- >200 million euros
  - If this: how much?

Does your hospital apply strategies to reduce prices and budget impact of expensive medicines [*Check all that apply*]

- - Parallel import
  - Parallel export
  - Group purchasing
  - Precision dosing: based on the weight of the patient even when a medicine has a fixed dose
  - Avoidance of medicine waste: collecting and re-dispensing unused oral drugs
  - Local production of medicines
  - Price (volume) negotiations for on medicines
    - For on patent medicines without competition: monopoly? (yes/no)
    - For on patent medicines with competition: oligopoly? (yes/no)
    - Price off patent medicines with competition: generic/biosimilar? (yes/no)
  - Other (please specify) ________________________
  - None of the above

1. Does your hospital have an financial incentive to negotiate lower medicine prices with companies?
   - No, there is no/little financial incentive
     - Financial gain obtained from negotiations are passed on to insurers
     - Financial gain obtained from negotiations passed on to government
   - Yes, there is an financial incentive, financial gain from negotiations is kept by your hospital
   - Other (please specify) ________________________
2. Do you think that the prices negotiated by your hospital are _______________compared to other hospitals in country?

- Higher
- Similar
- Lower

1. Do you think that the prices negotiated by your hospital are higher, similar or lower than prices in other European regions?

- _[higher/similar/lower]_ than in Northern Europe
- _[higher/similar/lower]_ than in Western Europe
- _[higher/similar/lower]_ than in Southern Europe
- _[higher/similar/lower]_ than in Eastern Europe

1. What is the value added tax (VAT) for cancer medicines in your country?

|  |
| --- |

1. Are there different VATs for types of medicines (for example intravenous/oral or generic/on patent)?

- Yes
- No

If yes,_please specify VAT differences:

|  |
| --- |

*Medicine specific questions*

In the following, we are asking you price and negotiation information for the following medicines:

| Palbociblib | Daratumumab | Abiraterone |
| --- | --- | --- |
| Ribociclib | Isatuximab | Lenalidomide |
| Abemaciclib | Nivolumab | Pomalidomide |
| Ibrutinib | Pembrolizumab | Gemcitabine |
| Acalabrutinib | Enzalutamide | Capecitabine |

**Please answer all questions B.1-8 with the perspective of your hospital.**

|  | Medicine | Palbociblib | Ribociclib | Abemaciclib | Ibrutinib | Acalabrutinib |
| --- | --- | --- | --- | --- | --- | --- |
| **Access** | ATC-code | L01EF01 | L01EF02 | L01EF03 | L01EL01 | L01EL02 |
|  | 1. Is this medicine **accessible** in your hospital?   *IF NO, do not proceed with questions 2-8 for that medicine.* | Yes/No | Yes/No | Yes/No | Yes/No | Yes/No |
| **Price information** | 2. What is the most purchased product for this active substance in your hospital (brand name, dosage, formulation, package size)? |  |  |  |  |  |
|  | - Brand name of most purchased product (for generics): | Ibrance | Kisqali | Verzenios | Imbruvica | Calquence |
|  | - Dosage and formulation of the most purchased product: | - Tablet   - 75mg   - 100mg   - 125mg - Capsule   - 75mg   - 100mg   - 125mg | - Tablet   - 200mg | - Tablet   - 50mg   - 100mg   - 150mg | - Tablet   - 140mg   - 280mg   - 420mg   - 560mg - Capsule   - 140mg | - Tablet   - 100mg - Capsule   - 100mg |
|  |  | Other:_____ | Other:_____ | Other:_____ | Other:_____ | Other:_____ |
|  | - Package size of the most purchased product: |  |  |  |  |  |
|  | 1. For this product (dosage, formulation, package) what is your hospital’s lowest purchasing price in 2022 local currency (including all discounts e.g. clawback, price/volume,…)? |  |  |  |  |  |
|  | 1. Is this price incl./excl. VAT? | incl./excl. | incl./excl. | incl./excl. | incl./excl. | incl./excl. |
| **Pricing context** | 1. Is this medicine priced differently for indications? | Yes/No | Yes/No | Yes/No | Yes/No | Yes/No |
|  | 1. Is this medicine imported from another country? | Yes/No | Yes/No | Yes/No | Yes/No | Yes/No |
|  | 1. Is your hospital negotiating the price for this product with pharmaceutical companies/wholesalers? | Yes/No | Yes/No | Yes/No | Yes/No | Yes/No |
|  | If yes:   - Is this purchased through a joint procurement group? | Yes/No | Yes/No | Yes/No | Yes/No | Yes/No |
|  | - Do interchangeable products (oligopoly) impact this negotiation? | Yes/No | Yes/No | Yes/No | Yes/No | Yes/No |
|  | - Do generic products impact this negotiation? | *Not applicable* | *Not applicable* | *Not applicable* | *Not applicable* | *Not applicable* |
|  | 1. Are you aware about further price arrangements for this medicine in your country that are not visible in the prices that you provided? | Yes/No | Yes/No | Yes/No | Yes/No | Yes/No |
|  | - If yes: on which level? (National/Insurer/Other) |  |  |  |  |  |

|  | Medicine | Daratumumab | Isatuximab | Nivolumab | Pembrolizumab | Enzalutamide |
| --- | --- | --- | --- | --- | --- | --- |
| **Access** | ATC-code | L01FC01 | L01FC02 | L01FF01 | L01FF02 | L02BB04 |
|  | 1. Is this medicine **accessible** in your hospital?  *IF NO, do not proceed with questions 2-8 for that medicine.* | Yes/No | Yes/No | Yes/No | Yes/No | Yes/No |
| **Price information** | 2. What is the most purchased product for this active substance in your hospital (brand name, dosage, formulation, package size)? |  |  |  |  |  |
|  | - Brand name of most purchased product (for generics): | Darzalex | Sarclisa | Opdivo | Keytruda | Xtandi |
|  | - Dosage and formulation of the most purchased product: | - Flacon 20mg/ml   - 5ml   - 15ml   - 20ml - Flacon 120mg/ml   - 15ml | - Flacon 20mg/ml   - 5ml   - 25ml | - Flacon 10mg/ml   - 4ml   - 10ml   - 12ml   - 24ml | - Flacon 25mg/ml   - 4ml | - Tablet   - 40mg - Capsule   - 40mg |
|  |  | Other:_____ | Other:_____ | Other:_____ | Other:_____ | Other:_____ |
|  | - Package size of the most purchased product: |  |  |  |  |  |
|  | 1. For this product (dosage, formulation, package) what is your hospital’s lowest purchasing price in 2022 local currency (including all discounts e.g. clawback, price/volume,…)? |  |  |  |  |  |
|  | 1. Is this price incl./excl. VAT? | incl./excl. | incl./excl. | incl./excl. | incl./excl. | incl./excl. |
| **Pricing context** | 1. Is this medicine priced differently for indications? | Yes/No | Yes/No | Yes/No | Yes/No | Yes/No |
|  | 1. Is this medicine imported from another country? | Yes/No | Yes/No | Yes/No | Yes/No | Yes/No |
|  | 1. Is your hospital negotiating the price for this product with pharmaceutical companies/wholesalers? | Yes/No | Yes/No | Yes/No | Yes/No | Yes/No |
|  | If yes:   - Is this purchased through a joint procurement group? | Yes/No | Yes/No | Yes/No | Yes/No | Yes/No |
|  | - Do interchangeable products (oligopoly) impact this negotiation? | Yes/No | Yes/No | Yes/No | Yes/No | Yes/No |
|  | - Do generic products impact this negotiation? | *Not applicable* | *Not applicable* | *Not applicable* | *Not applicable* | *Not applicable* |
|  | 1. Are you aware about further price arrangements for this medicine in your country that are not visible in the prices that you provided? | Yes/No | Yes/No | Yes/No | Yes/No | Yes/No |
|  | - If yes: on which level? (National/Insurer/Other) |  |  |  |  |  |

|  | Medicine | Abiraterone | Lenalidomide | Pomalidomide | Gemcitabine | Capecitabine |
| --- | --- | --- | --- | --- | --- | --- |
| **Access** | ATC-code | L02BX03 | L04AX04 | L04AX06 | L01BC05 | L01BC06 |
|  | 1. Is this medicine **accessible** in your hospital?  *IF NO, do not proceed with questions 2-8 for that medicine.* | Yes/No | Yes/No | Yes/No | Yes/No | Yes/No |
| **Price information** | 2. What is the most purchased product for this active substance in your hospital (brand name, dosage, formulation, package size)? |  |  |  |  |  |
|  | - Brand name of most purchased product (for generics): |  |  | Imnovid |  |  |
|  | - Dosage and formulation of the most purchased product: | - Tablet   - 250mg   - 500mg   - 1000mg | - Capsule   - 2.5mg   - 5mg   - 7.5mg   - 10mg   - 15mg   - 20mg   25mg | - Capsule   - 1mg   - 2mg   - 3mg   - 4mg | - Flacon100 mg/ml   - 2ml   - 10ml   - 15ml   - 20ml - Flacon 40 mg/ml   - 5ml   - 25ml   50ml | - Tablet   - 150mg   - 300mg   - 500mg |
|  |  | Other:_____ | Other:_____ | Other:_____ | Other:_____ | Other:_____ |
|  | - Package size of the most purchased product: |  |  |  |  |  |
|  | 1. For this product (dosage, formulation, package) what is your hospital’s lowest purchasing price in 2022 local currency (including all discounts e.g. clawback, price/volume,…)? |  |  |  |  |  |
|  | 1. Is this price incl./excl. VAT? | incl./excl. | incl./excl. | incl./excl. | incl./excl. | incl./excl. |
| **Pricing context** | 1. Is this medicine priced differently for indications? | Yes/No | Yes/No | Yes/No | Yes/No | Yes/No |
|  | 1. Is this medicine imported from another country? | Yes/No | Yes/No | Yes/No | Yes/No | Yes/No |
|  | 1. Is your hospital negotiating the price for this product with pharmaceutical companies/wholesalers? | Yes/No | Yes/No | Yes/No | Yes/No | Yes/No |
|  | If yes:   - Is this purchased through a joint procurement group? | Yes/No | Yes/No | Yes/No | Yes/No | Yes/No |
|  | - Do interchangeable products (oligopoly) impact this negotiation? | Yes/No | Yes/No | Yes/No | Yes/No | Yes/No |
|  | - Do generic products impact this negotiation? | Yes/No | Yes/No | *Not applicable* | Yes/No | Yes/No |
|  | 1. Are you aware about further price arrangements for this medicine in your country that are not visible in the prices that you provided? | Yes/No | Yes/No | Yes/No | Yes/No | Yes/No |
|  | - If yes: on which level? (National/Insurer/Other) |  |  |  |  |  |

**C. Participants perspective on participation**

1. What are barriers for you to share your data on prices? [*Check all that apply*]
   - Does not align with the hospital's strategic objectives
   - Do not believe that sharing price data can have positive societal effects on prices
   - Do not believe that sharing price data can have positive societal effects on access
   - Financial disadvantages in future negotiations with pharmaceutical companies
   - Financial disadvantages in future negotiations with health insurers/payers
   - Anti-competition laws applied in your country
   - Could have consequences based on contracts with pharmaceutical companies
   - Could have consequences based on contracts with health insurer/payers
   - Lack of resources or time constraints
   - Concerns about data privacy and security
   - Other (please specify) ________________________
2. What are facilitators for you to share your data on prices? [*Check all that apply*]

- Aligns with the hospital's strategic objectives
- Believe that sharing price data can have a positive societal effects on prices
- Believe that sharing price data can have a positive societal effects on access
- Financial advantages in future negotiations with pharmaceutical companies
- Financial advantages in future negotiations with health insurers/payers
- Facilitate collaboration, which is of interest of your hospital and future research
- Other (please specify) ________________________

1. Do you have any suggestions on how we could improve our study design or make it more appealing for participation in the future?

|  |
| --- |

**Thank you very much for participating in this study!**

# Appendix 4: Choosing the right comparator

To provide valuable insight in prices and price discrepancies, a reference price was chosen as comparator for the actual prices reported by hospitals. Different types of reference prices were reported in the IQVIA-MIDAS dataset^19^(Table 2). For the Netherlands, MIDAS data is derived from a second proprietary database from FarmInform^29^.

Table 2: Definitions of reference prices within the IQVIA-MIDAS dataset; *Definitions based on IQVIA MIDAS: Data Attributes, Measures and Statistics 2022

| **Reference price** | **Definitions*** |
| --- | --- |
| Manufacturer SellingPrice (List) * | Ex-factory price, or price to wholesaler (excludes sales tax) |
| Pharmacy Purchase Price (List)* | Ex-wholesaler price, trade price or price to chemist (excludes sales tax) |
| Retail Public Price (List)* | Pharmacy price, public price or price to patient (sales tax included or excluded depending on the country) |
| Price to hospital* | Hospital purchase price (does not correspond to tender price) -conceptual price that reflects the starting point for price negotiations between the hospital or ministry of health with the manufacturers or wholesalers |
| Trade price | Price based on the average sales revenue (pharmacy purchase price and wholesalers’ selling price) and sold units in a year across a country, excluding any confidential discounts |

In Figure 1, we illustrate the different types of references prices reported for Pembrolizumab (INF 25mg/ml 4ml) and Ribociclib (Tab 200mg x63). Depending on the country, the reference price can reflect a different distribution channel. For instance, in Germany, the 'Manufacturer Selling Price' is equivalent to the 'Price to Hospital,' whereas in the Netherlands, the 'Price to Hospital' is the same as the 'Pharmacy Purchase Price.' Furthermore, different types of reference prices might be reported depending on the product type (outpatient/inpatient) or formulation. For instance, in France, both the 'Price to Hospital' and the ‘Trade Price' were reported for Pembrolizumab (IV). For Ribociclib (oral), besides the ‘Trade Price,' the 'Manufacturer Selling Price List,' the 'Pharmacy Purchasing Price,' and the 'Retail Public Price’ were reported. Due to the variability, ‘Trade Price’ was calculated based on the average sales revenue (pharmacy purchase price and wholesalers’ selling price) and sold units within a year across a country, excluding any confidential discounts. By doing so, the ‘Trade Price’ takes the distribution structures of each country into account, ensuring comparability of the collected hospital prices across all countries and for all products. Thus, ‘Trade Price’ was used as a standardized reference price for the collected hospital prices.


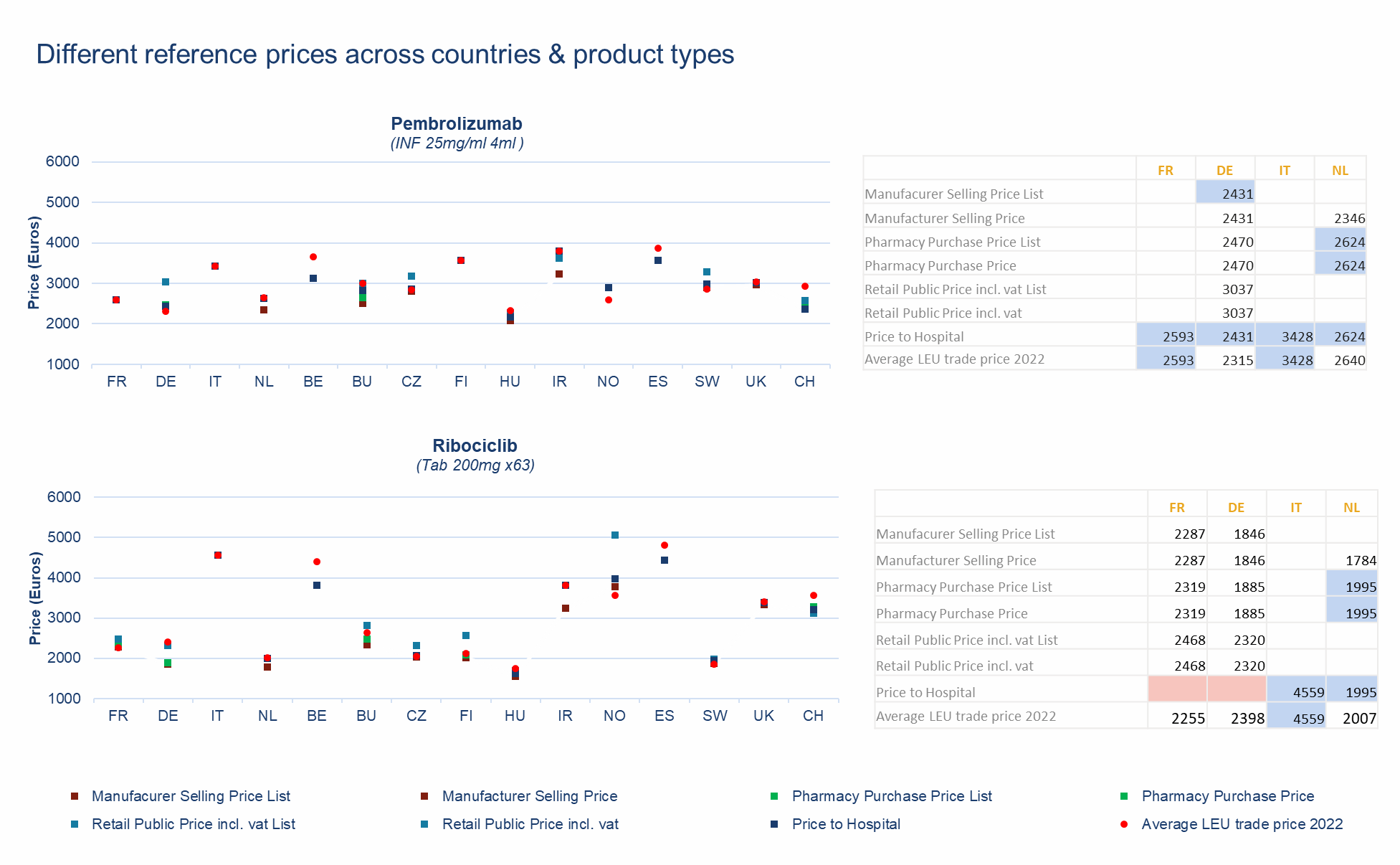


Figure 1: Different Reference prices across countries and product types

# Appendix 5: Price harmonization

To ensure that the comparison of prices across different dosages, formulations, and packaging remains valid and reliable, harmonization steps were implemented (if required) to ensure comparability. Based on all collected price points per product across countries, the most popular dosage, package size and formulation was identified so the harmonization steps was needed as limited as possible:

$\frac{Actual Price A}{Ref price A}=\frac{Product Price B}{Ref price B}$ 🡺 $Product price B =\frac{Ref price B *Actual Price A}{Ref price A}$

| *Actual Price A* | *The price collected for a medicine (Formulation A, Dosage A)* |
| --- | --- |
| *Ref price A* | *National reference price of a medicine (Formulation A, Dosage A)* |
| *Ref price B* | *National reference price of a medicine (Most popular Formulation B, Dosage B)* |
| *Product price B* | *Product price of a medicine (Most popular Formulation B & Dosage B)* |

If only the package size is different, the product price will be calculated without a reference price. The reason for this is that some countries solely report reference prices per tablet irrespective of the package size.

An example of harmonization of prices for a medicine with fictive data:

| Medicine | Collected Formulation, dosage package | Actual Price A | ref price A | Most popular  formulation, dosage, package | ref price B | Harmonization needed | Product price |
| --- | --- | --- | --- | --- | --- | --- | --- |
| Medicine 1 | Tab 125mg x 21 | 120 | 150 | Tab 125mg x 21 | 150 | No | 120 |
| Medicine 2 | Tab 100mg x 21 | 100 | 125 | Tab 125mg x 21 | 150 | Dosage  *(=(100*150)/125* | 120 |
| Medicine 3 | Tab 125mg x 63 | 365 | 400 | Tab 125mg x 21 | 150 | Package size  (=365/63*21) | 121.6 |

This harmonization method using the ratio of actual and reference prices was chosen as the dosage of some medicines is not always linear. For instance, the reference prices of Palbociclib are the same irrespective of the dosage and the reference price of Lenalidomide does not duplicate if the dosage does (Table 2).

| Product | Reference price _Netherlands_ | Reference price_Germany_ |
| --- | --- | --- |
| Palbociclib (Tab 75mg x 21) | 1976 | 1962 |
| Palbociclib (Tab 125mg x 21) | 1976 | 1962 |
| Lenalidomide (Tab 10 mg x 21) | 5023 | 3489 |
| Lenalidomide (Tab 25 mg x 21) | 5513 | 3633 |

# Appendix 6: Data analysis

- **Definitions**

Table 1: Definitions of the used terminology

| Term | Definitions |
| --- | --- |
| Product | The product (package size, dosage and formulation) were most price points were collected for, ensuring the least harmonization calculations needed. |
| Actual price | The actual price the hospital reports to be paying for a medicine. It can include confidential discounts negotiated by them or even at other levels (including national negotiations) |
| Product price | In order to harmonize the actual prices across all package sizes, formulations and dosages, the actual price was recalculated to the product price with a fixed package size, dosage and formulation |
| Reference price_National_ | The trade price based on the average sales revenue (pharmacy purchase price and wholesalers’ selling price) and sold units in a year across a country, excluding any confidential discounts. (IQVIA-MIDAS) |
| Reference price _AllCountries_ | The average of national reference prices of all included countries for the most used package |
| Discount | The discount calculated using the actual price and its reference price. |

- **Reporting product prices and their absolute and relative differences**

In our paper, Table 2 presents the product prices and their respective reference prices alongside their relative and absolute differences at both national and cross-country level. Figure 2 compares the national product prices with the average reference price across all countries, enabling an international comparison of prices.

$${Product price}_{National} \left( € \right)=\frac{\sum{Product prices}_{within a country} \left( € \right)}{N_{Product prices within a country}}$$

$$Absolute difference\left( € \right)= Highest product price \left( € \right)- Lowest product price(€)$$

$$Relative difference (\%) = \frac{\text{Absolute difference (€)}}{\text{ Product price(€) }}*100$$

- **Reporting discounts**

In our paper, Figure 3 reports discounts aggregated at country and product level (patent status, competition status and ATC-4 level).

$${Discount}\left( \% \right)=\left( 1-\frac{Actual price}{National ref price} \right)*100$$

- **Reporting price levels within a hospital**

In our paper, figure 4 demonstrates the hospitals’ perceived price levels (lower, similar, higher) compared to the reported price levels within their country or region.

$${Reported price level}_{Hospital} \left( \% \right)=\bar{x}_{hospital}( \frac{Actual price \left( € \right)}{{Product price}_{\frac{National}{Region}} \left( € \right)})$$

A difference of 5% from the average price level was chosen as a cut-off point for the similar price level.

Appendix 7: Hospitals’ perceived price levels compared to the reported price levels within their European region

Figure 2 demonstrates the hospitals’ perceived price levels compared to the reported price levels within their European region. Of the 22 hospitals included in this analysis (at least two hospitals included per region), 15 hospitals had an incorrect perception. Eleven hospitals perceived they had a lower prices compared to other hospitals within their region. Six of these hospitals were incorrect. Of those, four hospitals in the Netherlands and a hospital one Germany had higher price levels up to 30% compared to their regional average. Eleven hospitals thought they had a similar price compared to other hospitals. Nine of these hospitals were incorrect. Two hospitals in Serbia and the hospital in Southern Country 1 had higher price levels up to 18% compared to their regional average. Of all the hospitals included in this analysis, none of the hospitals thought they had a higher level compared to others within their EU Region.


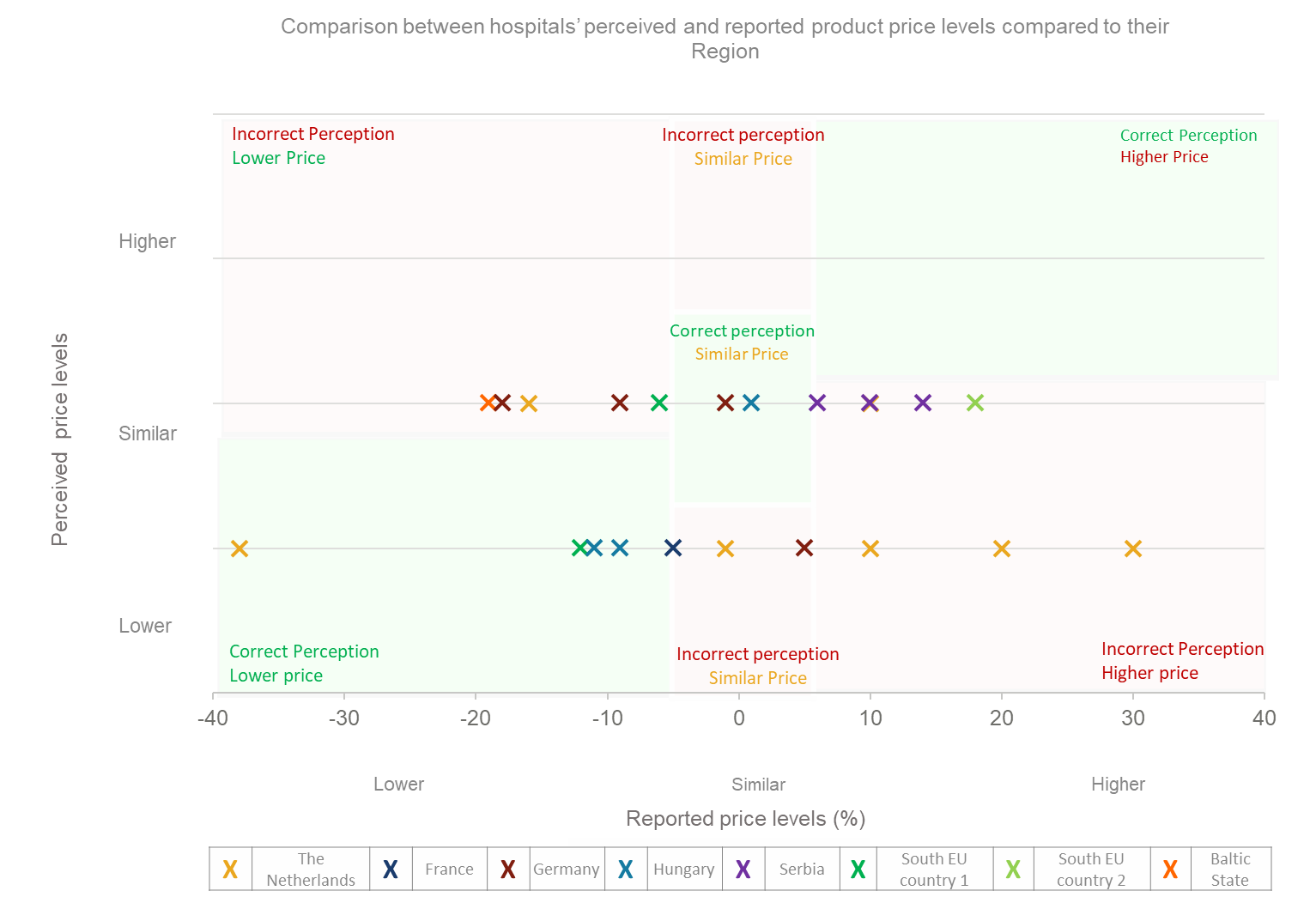


Figure 2: Hospitals’ perceived price levels compared to the reported price levels within their European region
